# AGHE PROGRAM OF MERIT: HEALTH PROFESSIONS PROGRAMS OPPORTUNITIES

**DOI:** 10.1093/geroni/igz038.2114

**Published:** 2019-11-08

**Authors:** Marilyn R Gugliucci, Donna Weinreich

**Affiliations:** 1 University of New England College of Osteopathic Medicine, Biddeford, Maine, United States; 2 Western Michigan State University, Kalamazoo, Michigan, United States

## Abstract

In 2015, the Program of Merit was expanded and adapted to implement a voluntary evaluation process for health professions programs that are choosing to integrate gerontology/geriatrics competencies in order to prepare students for working with older adults as well as their informal care partners. These programs are now eligible to apply for the Program of Merit designation. The Program of Merit for Health Professions Programs is based on the AGHE Standards and Guidelines for Gerontology/ Geriatrics in Higher Education, Sixth Edition (2015), specifically Chapters 11 and 12. This session will provide the information and support systems in place for health professions programs to be recognized with the AGHE Program of Merit designation.

